# Urinary albumin creatinine ratio associated with postoperative delirium in elderly patients undergoing elective non‐cardiac surgery: A prospective observational study

**DOI:** 10.1111/cns.13717

**Published:** 2021-08-20

**Authors:** Hui‐Lian Guan, He Liu, Xiao‐Yi Hu, Mannan Abdul, Ming‐Sheng Dai, Xing Gao, Xue‐Fen Chen, Yang Zhou, Xun Sun, Jian Zhou, Xiang Li, Qiu Zhao, Qian‐Qian Zhang, Jun Wang, Yuan Han, Jun‐Li Cao

**Affiliations:** ^1^ Jiangsu Province Key Laboratory of Anesthesiology & NMPA Key Laboratory for Research and Evaluation of Narcotic and Psychotropic Drugs Xuzhou Medical University Xuzhou City China; ^2^ Department of Anesthesiology The Affiliated Hospital of Xuzhou Medical University Xuzhou City China; ^3^ Department of Anesthesiology The First Affiliated Hospital of Bengbu Medical College Bengbu City China; ^4^ Department of Anesthesiology The Affiliated Huzhou Hospital Zhejiang University School of Medicine Huzhou Central Hospital Huzhou City China; ^5^ School of Public Health Shanghai University of Traditional Chinese Medicine Shanghai China; ^6^ Department of Anesthesiology Eye & ENT Hospital of Fudan University Shanghai China

**Keywords:** delirium, elderly patient, the blood‐brain barrier, urinary albumin creatinine ratio

## Abstract

**Introduction:**

The blood‐brain barrier (BBB) disruption contributes to postoperative delirium, but cost‐effective and non‐invasive assessment of its permeability is not practicable in the clinical settings. Urine albumin to creatinine ratio (UACR), reflecting systemic vascular endothelial dysfunction, may be a prognostic and predictive factor associated with postoperative delirium. The aim was to analyze the relationship between UACR and postoperative delirium in elderly patients undergoing elective non‐cardiac surgery.

**Materials and methods:**

Through stratified random sampling, a cohort of 408 individuals aged 60 years and older scheduled for elective non‐cardiac surgery were included between February and August 2019 in the single‐center, prospective, observational study. The presence of delirium was assessed using the Confusion Assessment Method (CAM) or Confusion Assessment Method for the ICU (CAM‐ICU) on the day of surgery, at 2 h after the surgery ending time and on the first 3 consecutive days with repeated twice‐daily, with at least 6‐h intervals between assessments. Urine samples were collected on one day before surgery, and 1st day and 3rd day after surgery. The primary outcome was the presence of postoperative delirium, and association of the level of UACR with postoperative delirium was evaluated with unadjusted/adjusted analyses and multivariable logistic regression.

**Results:**

Postoperative delirium was observed in 26.75% (107 of 400) of patients within 3 days post‐surgery. UACR‐Pre (OR, 1.30; 95% CI, 1.14–1.49, *p* < 0.001), UACR‐POD1 (OR, 1.20; 95% CI, 1.13–1.27, *p* < 0.001), and UACR‐POD3 (OR, 1.14; 95% CI, 1.08–1.20, *p* < 0.001) between the delirium and non‐delirium groups show a significant difference, even after adjusting for age, education levels, and other factors.

**Conclusion:**

As the marker of endothelial dysfunction, the high perioperative UACR value may be linked to the postoperative delirium in elderly patients undergoing elective non‐cardiac surgery.

## Graphical abstract

The blood‐brain barrier (BBB) disruption contributes to postoperative delirium, and urine albumin to creatinine ratio (UACR), reflecting systemic vascular endothelial dysfunction. As the marker of endothelial dysfunction, the high perioperative UACR value may be linked to the postoperative delirium in elderly patients undergoing elective non‐cardiac surgery.

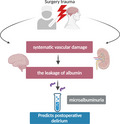

## 1 INTRODUCTION

Postoperative delirium has been reported in 10%–70% of all elderly surgical patients.^1^, ^2^, ^3^ It is associated with increased mortality, more extended hospital stay, reduced functional abilities,^4^, ^5^ long‐term cognitive dysfunction,^6^ and even dementia.^7^, ^8^ The symptoms of delirium often fluctuate throughout the day, and hypoactive delirium, characterized by inactivity and abnormal drowsiness, is likely to be missed. Thus, it is helpful to find the biomarkers for the earliest detection and monitoring of the presence of delirium.

It is well shown that BBB disruption can be characterized as the leakage of albumin in brain tissue, which progressively contributes to the pathophysiology of postoperative neurocognitive disorder in animal models.^9^, ^10^, ^11^ As the feasibility of obtaining brain tissue for albumin detection is highly invasive and compromising, there is lack of direct shreds of evidence that can resolve the mystery regarding the association between BBB damage and delirium in clinical settings. However, the determination of albumin in the urine is simple, non‐invasive, and economical. Strikingly, Ito et al^12^ proposed very interesting “strain vessel hypothesis” as a possible mechanism for cerebrovascular‐kidney connection, as both brain and kidneys are low‐resistance terminal organs that are exposed to high‐volume blood flow,^13^ and have hemodynamic similarities in the vascular beds. It is reported that microalbuminuria, as a marker for systematic vascular damage, is a common risk factor of dementia, kidney, and cardiovascular diseases.^14^, ^15^ Moreover, pre‐existing positive albuminuria is linked to postoperative acute kidney injury^16^, ^17^ and even indicates higher mortality of patients in intensive care unit (ICU).^18^

These evidences combined with the strain vessel hypothesis have given us an idea that perioperative albuminuria is related to postoperative delirium. The severity of albuminuria is defined by the urine albumin to creatinine ratio (UACR).^19^ Thus, in the present study, we propose the hypothesis that elderly patients, undergoing non‐cardiac surgery with a higher level of UACR, have a higher risk of postoperative delirium. The findings may have potential utility for screening patients for increased risk and also helps in understanding the etiology of delirium.

## 2 MATERIALS AND METHODS

### 2.1 Study design

“Urinary Albumin Creatinine Ratio Associated with Postoperative Delirium in Elderly Patients undergoing Elective Non‐cardiac Surgery” (NCT03860714) is a single‐center, prospective, observational study.

### 2.2 Characteristics and participants

Non‐neurologically impaired, elderly patients (≥60 years of age), ASA physical status I‐III, who had been referred for major non‐cardiac, non‐neurological, and non‐urological surgery under general anesthesia and expected a hospital stay of ≥3 days, were screened and enrolled between February and August 2019. Patients were excluded according to the following exclusion criteria^20^: significant impairments of vision, hearing or motor skills, history of a clear neurological disease, liver or kidney dysfunction (such as severe hepatitis, pyelonephritis), severe trauma or surgical history within one year, history of severe physical illness and alcoholism, Mini‐Mental State Examination (MMSE) score <15, refused to consent.

### 2.3 Ethics considerations

The study was approved by the Ethical Committee of the Affiliated Hospital of Xuzhou Medical University (Certification No. XYFY2018‐KL091, Chairperson Prof Tie Xu), Jiangsu, China, on January 24, 2019. All procedures performed in the study involving human participants were in accordance with the ethical standards of the institutional and/or national research committee and with the 2013 Declaration of Helsinki.^21^ Written informed consent was obtained from all subjects participating in the trial.

### 2.4 Observation Index

The information of the enrolled patients was recorded by a specialist. Preoperative basic information includes age, gender, body mass index (BMI), hypertension, diabetes mellitus, current or previous history of smoking, alcohol intake, educational level, ASA status, Mini‐mental State Examination (MMSE), Charlson's comorbidity index (CCI), and instrumental activities of daily living scale (IADL),^22^ and laboratory values include hemoglobin (Hb), serum albumin, alanine aminotransferase (ALT), aspartate aminotransferase (AST), blood urea, and serum creatinine (SCREA). Intraoperative information includes the type of surgery and anesthesia, anesthetic maintenance, duration of surgery and anesthesia, estimated blood loss during surgery, total intraoperative infusion, intraoperative blood transfusion, and occurrence of intraoperative hypotension. Postoperative information included postoperative ICU admission, patient controlled analgesia use, postoperative comorbidity within 3 days,^23^ and length of stay in hospital after surgery.

### 2.5 Delirium assessment

First of all, delirium fluctuates in mental status, so multiple repeated evaluations are required. Rigorous methodologies were used in delirium assessment. In this trial, trained investigators assessed patients for delirium using the Confusion Assessment Method (CAM)^24^ or the Confusion Assessment Method for the Intensive Care Unit (CAM‐ICU)^25^ for patients who were unable to speak (eg, still intubated) in the intensive care unit. Patients were assessed for delirium 2 h after surgery on the surgery day and were repeated twice‐daily in the morning, afternoon, or evening for the first 3 postoperative days, with at least 6 h between twice assessments.^26^ The CAM and the CAM‐ICU scales are reliable tools and have been consistent with the Diagnostic and Statistical Manual of Mental Disorders, 5th edition diagnostic criteria for delirium. We measured the presence of perioperative delirium seven times in 3 days. Once the test was positive, the patient was defined as delirium. Secondly, in order to avoid subjective bias when the same person is tested twice, different questions would be chosen. Additionally, research staff asked the nurses, families, and medical records for evidence of delirium, including confusion, agitation, sedation, hallucinations, and delusions. The assessment of delirium was carried out by trained researchers who did not know the patient's perioperative urinary albumin level and did not participate in data entry and statistical analysis.

### 2.6 Urinary albumin and Creatinine determination

Urine samples were collected from the patient's first‐morning void urine at 1 day before surgery and at 1st day stat and at 3rd day after surgery. If the patient retains the catheter, we will let the patient empty the urine bag at 10:00 PM a day before and collect the urine sample at 7:00 AM the next morning. In general, urine samples were collected between 6:00 and 7:30 AM. Urinary albumin and creatinine levels were measured independently by the Laboratory of Affiliated Hospital of Xuzhou Medical University. Urinary albumin levels were measured by immunoturbidimetry using a full automatic protein analyzer (Siemens Healthcare Diagnostics), and urinary creatinine levels were performed by enzymatic method using Cobas 8000 (Roche Diagnostics); then, we calculated a ratio between them, which is known as the urine albumin/creatinine ratio (UACR).

### 2.7 Outcome definition

The primary outcome was the presence of postoperative delirium. The principal objective of this study was to assess the relationship between the UACR levels of 1 day before surgery and 1st, 3rd day after surgery and the presence of postoperative delirium. The secondary objective of this study was to define the change of UACR levels between delirium group and non‐delirium group at 3 time points during perioperative period. Moreover, this study was designed to determine the receiver operating characteristic (ROC) curve of the UACR at 3 time points and with the presence of postoperative delirium.

### 2.8 Sample size and Statistical analysis

Based on the previous literature^27^, ^28^ and preliminary data, we estimated that the incidence of postoperative delirium is 25%. The difference of UACR between delirium group and non‐delirium group was 4 mg/g, and the standard deviation (SD) was 10. Thus, 356 participants would provide 90% power and using a two‐tailed alpha of 0.05. Considering a drop‐out rate of approximately 10%, the total sample size was calculated as 400. Sample size calculations were performed using PASS, version 11 (NCSS).

Statistical analyses were carried out using SPSS, version 23.0 (IBM). The normality assumption was assessed using the Kolmogorov‐Smirnov test in all analyses. Continuous variables were presented as a mean (SD) or median (inter quartile range [IQR]), and categorical variables were presented as the number of patients (percentage [%]). Group comparisons were made using 2 independent sample *t* tests for continuous variables with a normal distribution, the Mann‐Whitney *U* test for continuous variables with a non‐normal distribution, or the chi‐square test or Fisher's exact test for dichotomous and categorical data. The forward likelihood ratio method was used in multivariable logistic regression. Assessing the multicollinearity between univariate values of *p* < 0.1, it was found that only duration of anesthesia and duration of surgery were significantly collinear (Pearson's correlation coefficient = 0.963, *p* < 0.001), and the variance inflation factor of duration of surgery was higher. Thus, duration of surgery was excluded, and the others were included in the following multivariable logistic regression model to determine independent risk factors for postoperative delirium. All hypothesis testing was 2 tailed. Except for the UACR levels at the three time points, *p* < .05 was considered to indicate significance. According to the Bonferroni correction, the UACR levels of 1 day before surgery and 1st, 3rd day after surgery were considered significant when *p* < 0.017. The urinary albumin to creatinine ratio data, which allowed for determination of whether patients developed postoperative delirium, was assessed using receiver operating characteristic (ROC) curve analysis. The optimal cutoff value was defined as the point of the urinary albumin to creatinine ratio data of 1st day exhibiting the highest sum of sensitivity and specificity. The area under the receiver operating characteristic curve is commonly interpreted as excellent (0.9–1), good (0.75–0.89), fair (0.6–0.74), low (0.5–0.59), or fail/no (<0.5) predictive ability. We conducted linear mixed effect model with random effect of the repeated measurement of UACR. The data were analyzed by SAS^®^ 9.2 Software (SAS Institute Inc., SAS Campus Drive). All hypothesis testing was 2 tailed. *p* < 0.05 was considered to indicate significance.

## 3 RESULTS

**FIGURE 1:**
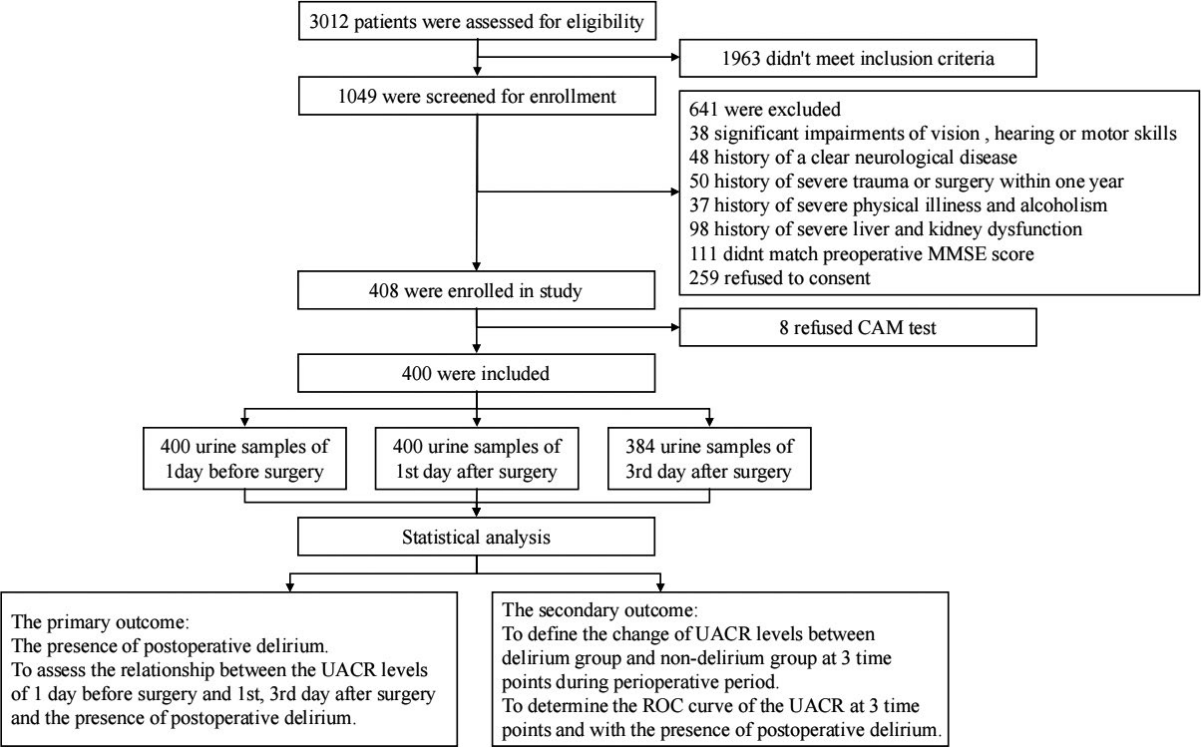
Enrollment flow chart for the study population

During the study period, 3012 patients were assessed for eligibility, of whom 1049 satisfied the inclusion criteria. Among those screened, 641 patients were excluded from the study for various reasons. In total, 408 qualified patients provided their written informed consent and were enrolled in the study. During the postoperative period, 8 patients refused delirium assessments. Thus, a total of 400 patients were included in the final data analyses. Including 2 patients with delirium assessment by nurse. And 16 patients had forgotten or failed to collect urine specimens at the specified time point on the 3rd day after surgery, resulting in 16 urine samples missing. By analyzing the presence of postoperative delirium and baseline data of these 16 patients, except for the statistically significant difference between genders, other indicators are balanced between the two groups. It shows that the overall data are still representative (Shown in Table S1A,B). Moreover, based on the principle of intentionality analysis, we conducted statistical analysis on the interpolation data, and the statistical analysis results were almost the same (Shown in Table S1C). A flow chart of patient enrollment in the study is shown in Figure 1.

### 3.1 Perioperative basic information

The baseline characteristics and laboratory biomarkers of delirium and non‐delirium groups, along with the results of the stated univariate analyses, are shown in Table 1. Patients who developed delirium were older (*p* < 0.001), lower education level (*p* = 0.020), preoperative Instrument Activities of Daily Living disability items ≥ 1 (*p* = 0.003), lower MMSE scores (*p* = 0.002), and lower hemoglobin concentrations (*p* = 0.033) were more prevalent in the group with postoperative delirium. Sex, BMI, smoking history, alcohol intake, hypertension, diabetes mellitus, ASA status, CCI, serum albumin, ALT, AST, blood urea, and SCREA were not significantly different between the delirium and non‐delirium groups.

**TABLE 1:** Characteristics the patients at baseline

	Delirium, *n* = 107	Non‐delirium, *n* = 293	*p*
Male [*n* (%)]	63 (58.88)	183 (62.46)	0.515
Age (year)	71 (66–76)	68 (64–72)	<0.001*
BMI (kg/m^2^)	23.67±3.97	23.84±3.37	0.674
Hypertension [*n* (%)]	30 (28.04)	100 (34.13)	0.250
Diabetes Melllitus [*n* (%)]	16 (14.95)	35 (11.95)	0.425
Ever a smoker [*n* (%)]	45 (42.06)	113 (38.57)	0.527
Alcohol intake [*n* (%)]	28 (26.17)	95 (32.42)	0.230
Cerebral [*n* (%)]	23 (21.50)	63 (21.50)	0.999
Educational level [*n* (%)]			
Illiterate	43 (40.19)	76 (25.94)	0.020*
Elementary or middle school	46 (42.99)	163 (55.63)	
High school and above	18 (16.82)	54 (18.43)	
ASA status [*n* (%)]			
Ⅰ–Ⅱ	88 (82.24)	256 (87.37)	0.191
Ⅲ	19 (17.76)	37 (12.63)	
MMSE (score)	24 (20–27)	26 (22.50–28)	0.002**
CCI (score)	2 (2–3)	2 (1–3)	0.051
IADL ≥ 1 [*n* (%)]	37 (34.58)	59 (20.14)	0.003**
Laboratory values			
Hemoglobin (g/L)	128 (110.50–139.25)	131.5 (120.25–143)	0.033*
Albumin (g/L)	41.55 (38.63–44.45)	42.6 (39.30–45.30)	0.092
ALT (U/L)	15 (11–23)	15 (11–22)	0.712
AST (U/L)	18 (15–22)	18 (15–22)	0.618
Blood Urea (mmol/L)	5.35 (4.22–6)	5.1 (4.19–6.11)	0.576
SCREA (μmol/L)	61 (53–71.50)	63 (54–71)	0.723

Note

*p* Values were calculated using chi‐square tests for dichotomous and ranked variables. For differences between means, *p* values were calculated with the unpaired *t* test. For differences between medians, *p* values were calculated with the Mann‐Whitney *U* test. *p < 0.05, **p < 0.01.

Abbreviations: ALT, Alanine aminotransferase; AST, Aspartate aminotransferase; BMI, Body mass index; CCI, Charlson Comorbidity Index; IADL, Instrumental activity of daily living; MMSE, Mini‐Mental Sate Examination; SCREA, Serum creatinine.

Intra‐ and postoperative factors of postoperative delirium are displayed in Table 2. Patients with delirium had a higher number of intraoperative blood transfusion (*p* = 0.033) and ICU admission (*p* < 0.001). In addition, patients with delirium had a longer stay in hospital after surgery (*p* = 0.004). In contrast, there were no differences in the type of surgery and anesthesia maintenance, combined nerve block, duration of surgery and anesthesia, estimated blood loss, total intraoperative infusion, the occurrence of hypotension, in the use of patient controlled analgesia, or postoperative complications within 3 days between patients whether or not delirium developed.

**TABLE 2:** Intraoperative and postoperative data

	Delirium, *n* = 107	Non‐delirium, *n* = 293	*p*
Sugery type [*n* (%)]			
Intra‐thoracic	39 (36.45)	105 (35.84)	0.737
Intra‐abdominal	20 (18.69)	65 (22.18)	
Spinal and extremital	48 (44.86)	123 (41.98)	
General plus nerve block [*n* (%)]	35 (32.71)	83 (28.33)	0.395
Anesthesia maintenance [*n* (%)]			
Propofol	3 (2.80)	4 (1.37)	0.630
Propofol plus Sevoflurane	90 (84.11)	261 (89.08)	
Etomidate plus Sevoflurane	4 (3.74)	5 (1.71)	
Etomidate plus Propofol and Sevoflurane	10 (9.35)	23 (7.85)	
Duration of surgery (min)	190 (135–240)	170 (130–225)	0.094
Duration of anesthesia (min)	235 (60–280)	200 (160–260)	0.082
Estimated blood loss during surgery (ml)	200 (100–300)	100 (100–200)	0.098
Total intraoperative infusion (ml)	2000 (1500–2300)	2000 (1500–2400)	0.881
Intraoperative blood transfusion [*n* (%)]	13 (12.15)	17 (5.80)	0.033*
Hopotension [*n* (%)]	10 (9.35)	18 (6.14)	0.266
ICU admission [*n* (%)]	34 (31.78)	36 (12.29)	<0.001**
PCA [*n* (%)]	82 (76.64)	211 (72.01)	0.355
Postoperative complications within 3 days [*n* (%)]	29 (27.10)	66 (22.53)	0.341
Length of stay in hospital after surgery (days)	11 (9–14)	10 (7–12)	0.004**

Note

*p* Values were calculated using chi‐square tests for dichotomous variables. For differences between medians, *p* values were calculated with the Mann‐Whitney *U* test. *p < 0.05, **p < 0.01.

Abbreviations: ICU, Intensive care unit; PCA, Patient‐controlled analgesia.

### 3.2 Primary outcome

Postoperative delirium was observed in 26.75% (107 of 400) of patients within 3 days post‐surgery. The univariate analysis identified that UACR‐Pre (OR, 1.38; 95% CI, 1.21–1.57, *p* < 0.001), UACR‐POD1 (OR, 1.24; 95% CI, 1.16–1.31, *p* < 0.001), and UACR‐POD3 (OR, 1.16; 95% CI, 1.10–1.22, *p* < 0.001) were statistically significant. The multivariable logistic regression analysis identified that UACR‐Pre (OR, 1.30; 95% CI, 1.14–1.49, *p* < 0.001), UACR‐POD1 (OR, 1.20; 95% CI, 1.13–1.27, *p* < 0.001), and UACR‐POD3 (OR, 1.14; 95% CI, 1.08–1.20, *p* < 0.001) remained independent predictors of the occurrence of postoperative delirium even after adjusting for influencing factors (*p* < 0.1). These were age, educational level, MMSE, CCI, hemoglobin, albumin, duration of anesthesia, estimated blood loss during surgery, intraoperative blood transfusion, IADL, and ICU admission (Table 3).

**TABLE 3:** Associations between the UACR and postoperative delirium

	Multivariable logistic regression analysis^a^	
	Odds Ratio (95% CI)	*p* Value
Model 1		
Age (year)	1.07 (1.03–1.11)	0.002
MMSE (score)	0.93 (0.87–0.99)	0.022
ICU admission [*n* (%)]	2.74 (1.52–4.92)	0.001
UACR‐Pre (10 mg/g)	1.30 (1.14–1.49)	<0.001
Model 2		
Age (year)	1.06 (1.01–1.10)	0.012
MMSE (score)	0.93 (0.87–0.99)	0.030
ICU admission [*n* (%)]	2.45 (1.33–4.52)	0.004
UACR‐POD1 (10 mg/g)	1.20 (1.13–1.27)	<0.001
Model 3		
Age (year)	1.06 (1.01–1.10)	0.009
MMSE (score)	0.910 (0.85–0.97)	0.004
ICU admission [*n* (%)]	2.63 (1.42–4.86)	0.002
UACR‐POD3 (10 mg/g)	1.14 (1.08–1.20)	<0.001

Note

The UACR data on 1 day before surgery, 1st and 3rd day after surgery were entered into Logistic regression. Forward LR method was used in multivariable logistic regression column. Variables that were significant in univariate analyses (*p* < 0.1) were included in a multivariate Logistic regression model to determine the risk‐adjusted predictors of postoperative delirium.

Abbreviations: CCI, Charlson Comorbidity Index; CI, Confidence interval; IADL, Instrumental activity of daily living; ICU, Intensive care unit; LR, Likelihood ratio; MMSE, Mini‐Mental Sate Examination; POD, Postoperative day; Pre, Preoperative; UACR, Urine albumin to creatinine ratio.

^a^ Occurrence of postoperative delirium was modeled as a function of all predictors. Multivariate logistic regression analysis was performed by using a Forward LR procedure.

### 3.3 Secondary outcomes

Urine albumin level is presented as a continuous variable. The UACR levels of delirium and non‐delirium groups on 1 day before surgery and 1st day stat and 3rd day after surgery are shown in Table 4. According to Type 3 tests of fixed effects in mixed effect model, there is significance in the interaction between group and time (*p* < 0.0001). Compared to baseline of the non‐delirium, the UACR mean difference of from 1 day after surgery to baseline (25.3, 95%CI: 13.8–36.9) of POD increased significantly (*p* < 0.0001). The same results showed in the difference from 3 day after surgery to baseline (36.7, 95% CI: 26.8–46.6).

**TABLE 4:** Comparison UACR of 3 time points between two groups

Time	Group		Difference	
	Non‐delirium	Delirium	∆ (95%CI)	*p* Value
Baseline (UACR‐Pre)	18.5 ± 13.7	36.8 ± 42.7	Ref	—
UACR‐POD1	39.2 ± 31.6	94.3 ± 85.3	25.3(13.8, 36.9)	<0.0001
UACR‐POD3	37.3 ± 35.0	82.4 ± 92.8	36.7(26.8, 46.6)	<0.0001

Abbreviations: POD, Postoperative day; Pre, Preoperative; UACR, Urinary albumin to creatinine ratio.

**FIGURE 2:**
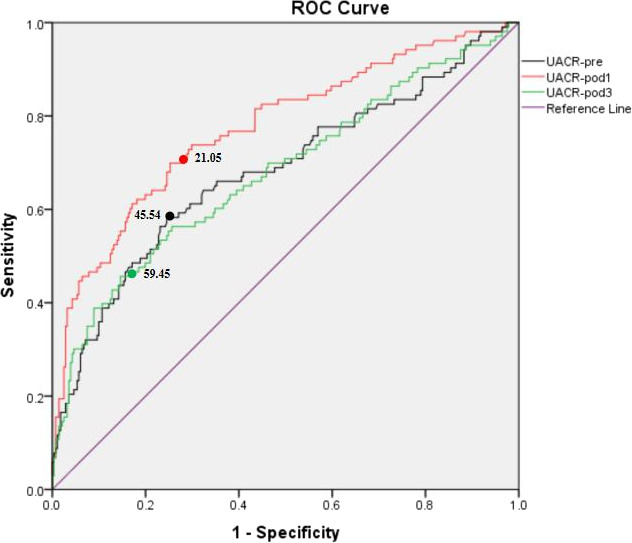
ROC curve of UACR levels. Abbreviations: POD, Postoperative day; Pre, Preoperative; ROC, receiver operating characteristic; UACR, Urinary albumin to creatinine ratio

Receiver operating characteristic curve was created for the UACR levels (Figure 2). The area under the curve (AUC) for UACR‐Pre was 0.68 (95% CI, 0.62–0.75; *p* < 0.001). However, the AUC value of UACR‐Pre was fair. The optimal cutoff value was found to be 21.05 with a sensitivity of 57.01% and specificity of 75.09%. The AUC for UACR‐POD1 was 0.76 (95% CI, 0.56–0.88; *p* < 0.001). The optimal cutoff value was found to be 45.54 with a sensitivity of 68.22% and specificity of 73.72%. The AUC for UACR‐POD3 was 0.68 (95% CI, 0.61–0.74; *p* < 0.001). The optimal cutoff value was found to be 59.45 with a sensitivity of 45.63% and specificity of 85.41%. The AUC value of UACR‐POD1 was good, and the AUC value of UACR‐POD3 was fair.

## 4 DISCUSSION

The present study demonstrated that the perioperative level of UACR to be the independent predictor of delirium in the elderly population after elective non‐cardiac surgery. The most important finding of the study is that the perioperative UACR value is highly associated with the presence of postoperative delirium. The area under the ROC curve for UACR‐Pre was 0.68, UACR‐POD1 was 0.76, and UACR‐POD3 was 0.68.

As an important antecedent of microalbuminuria, endothelial dysfunction is not only linked to cognitive decline, but also more often as a well‐known contributor to the pathophysiology of cardiovascular disease, including hypertension, coronary artery disease, diabetes, and chronic renal failure.^18^, ^29^ It may partly explain the reason why the preoperative UACR value can only provide a fair predictive ability for delirium. As albuminuria is more often relevant among the patients with the history of kidney disease, which is an interfering factor in the study, so we excluded patients with renal dysfunction before surgery.

The vascular endothelium is sensitive to mechanical stimuli like surgery stress and hormonal stimuli from vasoactive substances. It is well‐documented that surgery trauma can induce acute endothelial dysfunction, characterized as the appearance of increased albumin, or increased level of Matrix metallopeptidase 9, in rodents' brain tissue.^30^ A clinical study found that thoracic surgery trauma is related to postoperative appearance of microalbuminuria, which is an early identification of complication risks.^31^ Albuminuria also predicts illness severity in critically ill patients among critical patients.^32^, ^33^ In line with these findings, we also found that surgery trauma itself can cause the new appearance of albuminuria, which indicates the acute endothelial dysfunction. Moreover, UACR value at 1 day after surgery provides a better predictive ability for delirium than preoperative one. Thus, continually monitoring perioperative UACR may be helpful for predicting or early detecting delirium, especially for the hypoactive type. However, as the area under the ROC curve is not high, it would be better to combine several other biomarkers to reduce the rate of missed delirium diagnoses and then enhance the specificity and sensitivity.

Other etiology has been found underlying the development of POD. Dysfunctional cerebral autoregulation affects cerebral perfusion and oxygenation, which may expose the brain to the subsequent risk of developing delirium.^34^ Burst suppression induced by anesthetics may damage neuronal, vascular, and mitochondrial function, thereby promoting neurological complications such as delirium.^35^ The interaction between intestinal microbes and the brain is bilateral through the microbiome‐gut‐brain axis and may play a role in neurodegenerative diseases.^36^, ^37^ However, the mechanism of delirium is still unclear. This study only explored the correlation between UACR level and postoperative delirium based on the cerebrovascular‐kidney connection theory.

Although delirium is an acute brain complication that occurs after surgery, it is reported to be related to the presence of postoperative cognitive dysfunction (POCD).^6^ As continuous monitoring of UACR is simple, cost‐effective, and non‐invasive, the relationship between the UACR and POCD is worth researching. Particularly, urinary albumin is a predictor for several kinds of cognitive impairment. Shoko et al^38^ found that cognitive function was negatively correlated with microalbuminuria in the population of community‐dwelling Japanese. H.‐K. et al^39^ has found that the presence of microalbumin or high levels of urinary albumin excretion is inversely associated with cognitive function. Laura et al^40^ performed an 11‐year follow‐up study of 8028 subjects and finally presented that microalbuminuria and large amounts of albuminuria were predictors of cognitive performance in the general population.

Besides the value of UACR, age, baseline MMSE level, and ICU admission were also independent risk factors of postoperative delirium, which are all reported to be the well‐recognized risk factors of postoperative delirium. With the increase of age in elderly patients, their neuronal apoptosis increased, brain tissue degeneration and atrophy, and the decrease in central neurotransmitter content were related to postoperative delirium.^41^ The MMSE score is primarily used to assess mild cognitive impairment (MCI), and elderly MCI patients are associated with the incidence of postoperative delirium.^42^ Due to the analgesic sedatives commonly used (eg, opioids and benzodiazepines), adverse environmental factors, mechanical ventilation related sleep deprivation, or sleep disruption in patients, ICU admission is highly related to the present of postoperative delirium.^43^, ^44^

The present study has several advantages. First, it has shown a beneficial aspect compared with other biomarkers that are related to delirium and their application in clinical practice is hampered by invasive sample collection and complicated diagnostic procedures. On the other hand, urine samples are non‐invasive procedure and easy to be collected by participants and associated with minimal risk. Secondly, UACR level was monitored 3 times during the perioperative period (preoperative, 1st and 3rd day after surgery), which clearly showed the surgery stress induced UACR fluctuations during perioperative period. Finally, to our knowledge, it is one of the first domestic study regarding relationship between UACR and the presence of postoperative delirium.

Several limitations to this study should be considered in interpreting these results. Firstly, the severity of postoperative delirium and the time when it first appeared were not recorded, which could have helped in exploring the severity of postoperative delirium and UACR levels. Secondly, although we measured the presence of perioperative delirium 7 times in 3 days, it has been suggested that the delirium measurement could be conducted until the 5th day after surgery. However, postoperative delirium mainly occurs within 3 days after surgery, and the percentage is about 80%–90%.^20^, ^45^ Thirdly, we have not classified the form of postoperative delirium separately. A rough distribution of delirium presentations suggests that it may be of the hyperactive form in 25%, hypoactive in 50%, and mixed in 25% of cases. Since the hypoactive delirium is more difficult to be detected early, patients with postoperative hypoactive delirium were may classified the non‐delirium groups. For the assessment of delirium, objective indicators are needed for diagnosis or auxiliary diagnosis. Therefore, exploring markers in the future is valuable for discovering hypoactive delirium. We hope that this study will have a certain enlightening effect.

## 5 CONCLUSION

As the marker of endothelial dysfunction, perioperative new onset of microalbuminuria may be linked to the postoperative delirium. UACR monitoring during the perioperative period is low‐cost, easily conducted, and non‐invasive. This study offered the first line evidence that the increased perioperative UACR is related to the incidence of postoperative delirium among the elderly patients. Further study is still needed to understand this potential relationship, and long‐term follow‐up is required to focus on the long‐term prognosis of these patients with higher level of UACR.

## CONFLICT OF INTEREST

The authors declare that they have no conflicts of interest.

## AUTHOR CONTRIBUTIONS

Jun‐Li Cao designed the study, critical review of the manuscript, approves the final version, and is accountable for the work. Hui‐Lian Guan, He Liu, and Yuan Han designed the study, conducted the study, collected the data, prepared the manuscript, and critical review of the manuscript and approve the final version. They are accountable for the work. Ming‐Sheng Dai, Xing Gao, Yang Zhou, and Jian Zhou helped conduct the study and collected the data. Xiao‐Yi Hu, Xue‐fen Chen, and Xun Sun analyzed and interpreted the data. Qiu Zhao and Xiang Li helped test samples and analyzed the data. Qian‐Qian Zhang, Mannan‐Abdul, and Jun Wang helped prepare the manuscript and critical review of the manuscript.

## Supporting information

Table S1

## Data Availability

The data that support the findings of this study are available from the corresponding author upon reasonable request.
