# Supplementary material for: Urinary albumin creatinine ratio associated with postoperative delirium in elderly patients undergoing elective non‐cardiac surgery: A prospective observational study
Source: CNS Neurosci Ther. 2021 Aug 20;28(4):521–30. doi: 10.1111/cns.13717 (PMC8928921; doi:10.1111/cns.13717)
Supplement: Supplementary file 1 — Table S1 [file CNS-28-521-s001.doc]

**Table A. Characteristics the Patients at Baseline**

|  | Missing Data,  n = 16 | Remaining Data,  n = 384 | *P* |
| --- | --- | --- | --- |
| Male [n (%)] | 4 (25.00) | 240 (62.50) | 0.003* |
| Age (year) | 69.5 (66.25-73.5) | 68 (64-73) | 0.647 |
| BMI (kg/m2) | 23.94 (19.73-25.33) | 23.75 (21.46-25.99) | 0.770 |
| Hypertension [n (%)] | 6 (37.50) | 124 (32.29) | 0.663 |
| Diabetes Melllitus [n (%)] | 3 (18.75) | 48 (12.50) | 0.725 |
| Ever a smoker [n (%)] | 5 (31.25) | 153 (39.84) | 0.491 |
| Alcohol intake [n (%)] | 3 (18.75) | 120 (31.25) | 0.432 |
| Cerebral [n (%)] | 4 (25.00) | 82 (21.35) | 0.970 |
| Educational level [n (%)] |  |  | 0.726 |
| Illiterate | 6 (37.50) | 113 (29.43) |  |
| Elementary or middle school | 8 (50.00) | 201 (52.34) |  |
| High school and above | 2 (12.50) | 70 (18.23) |  |
| ASA status [n (%)] |  |  | 0.586 |
| Ⅰ - Ⅱ | 15 (93.75) | 329 (85.68) |  |
| Ⅲ | 1 (6.25) | 55 (14.32) |  |
| MMSE (score) | 24 (20.5-25.75) | 25 (22-28) | 0.133 |
| CCI (score) | 2 (2- 3) | 2 (1- 3) | 0.181 |
| IADL≥1 [n (%)] | 3 (18.75) | 93 (24.22) | 0.839 |
| Laboratory values |  |  |  |
| Hemoglobin (g/L) | 122.5 (118.5-136.25) | 131 (118-142) | 0.151 |
| Albumin (g/L) | 42 (39.55-45.15) | 42.35 (39.08-45) | 0.938 |
| ALT (U/L) | 18 (12-20) | 15 (11-22) | 0.952 |
| AST (U/L) | 16 (15-20.75) | 18 (15-22) | 0.684 |
| Blood Urea (mmol/L) | 5.1 (4.20-5.62) | 5.17 (4.2-6.1) | 0.787 |
| SCREA (μmol/L) | 58.5 (53.25-65.50) | 62 (54-71) | 0.289 |

**Table B. Intraoperative and Postoperative Data**

|  | Missing Data,  n = 16 | Remaining Data,  n = 384 | *P* |
| --- | --- | --- | --- |
| Sugery type [n (%)] |  |  | 0.635 |
| Intra-thoracic | 5 (31.25) | 139 (36.20) |  |
| Intra-abdominal | 5 (31.25) | 80 (20.83) |  |
| Spinal and extremital | 6 (37.50) | 165 (42.97) |  |
| General plus nerve block [n (%)] | 1 (6.25) | 117 (30.47) | 0.072 |
| Anesthesia maintenance [n (%)] |  |  | 0.654 |
| Propofol | 0 (0.00) | 7 (1.82) |  |
| Propofol plus Sevoflurane | 14 (87.50) | 337 (87.76) |  |
| Etomidate plus Sevoflurane | 0 (0.00) | 9 (2.34) |  |
| Etomidate plus Propofol and Sevoflurane | 2 (12.50) | 31 (8.07) |  |
| Duration of surgery (min) | 170 (130-303.75) | 175 (130-230) | 0.237 |
| Duration of anesthesia (min) | 130 (102.50-256.25) | 210 (161.25-265) | 0.176 |
| Estimated blood loss during surgery (ml) | 100 (100-200) | 100 (100-200) | 0.901 |
| Total intraoperative infusion (ml) | 1750 (1500-2450) | 2000 (1500-2375) | 0.769 |
| Intraoperative blood transfusion [n (%)] | 2 (12.50) | 28 (7.29) | 0.771 |
| Hopotension [n (%)] | 1 (6.25) | 27 (7.03) | 0.903 |
| ICU admission [n (%)] | 4 (25.00) | 66 (17.19) | 0.638 |
| PCA [n (%)] | 12 (75.00) | 281 (73.18) | 0.871 |
| Postoperative complications within 3 days [n (%)] | 4 (25.00) | 91 (23.70) | 0.905 |
| Length of stay in hospital after surgery (d) | 10.5 (7.75-12.75) | 10 (7-13) | 0.615 |
| Postoperative delirium [n (%)] | 4 (25.00) | 103 (26.82) | 1.000 |
| UACR-Pre (mg / g) | 14.52 (12.49-23.18) | 16.74 (11.01-24.83) | 0.726 |
| UACR-POD1 (mg / g) | 30.45 (15.79-55.70) | 37.87 (23.64-60.86) | 0.202 |

**Table C. Associations between the UACR and Postoperative Delirium (impute missing data of 16 urine samples)**

|  | Multivariable logistic regression analysis^b^ | |
| --- | --- | --- |
|  | Odds Ratio (95% CI) | *P* value |
| Model 1 |  |  |
| Age (year) | 1.07(1.03-1.11) | 0.002 |
| MMSE (score) | 0.93(0.87-0.99) | 0.022 |
| ICU admission [n (%)] | 2.74(1.52-4.92) | 0.001 |
| UACR-Pre (10 mg /g ) | 1.30(1.14-1.49) | <0.001 |
| Model 2 |  |  |
| Age (year) | 1.06(1.01-1.10) | 0.012 |
| MMSE (score) | 0.93(0.87-0.99) | 0.030 |
| ICU admission [n (%)] | 2.45(1.33-4.52) | 0.004 |
| UACR-POD1 (10 mg /g ) | 1.20(1.13-1.27) | <0.001 |
| Model 3 |  |  |
| Age (year) | 1.06(1.01-1.10) | 0.013 |
| MMSE (score) | 0.910(0.85-0.97) | 0.006 |
| ICU admission [n (%)] | 2.67(1.47-4.84) | 0.001 |
| UACR-POD3 ( 10 mg /g ) | 1.12(1.07-1.18) | <0.001 |
